# Contribution of TLR4 to colorectal tumor microenvironment, etiology and prognosis

**DOI:** 10.1007/s00432-022-04199-4

**Published:** 2022-07-16

**Authors:** Elise E. Crame, Saeed Nourmohammadi, Hannah R. Wardill, Janet K. Coller, Joanne M. Bowen

**Affiliations:** 1 Discipline of Physiology, School of Biomedicine, The University of Adelaide, Level 2 Helen Mayo South, North Terrace, Adelaide, SA 5000 Australia; 2 Discipline of Pharmacology, School of Biomedicine, The University of Adelaide, Adelaide, SA Australia; 3 Precision Medicine (Cancer), The South Australian Health and Medical Research Institute, Adelaide, SA Australia

**Keywords:** Toll-like receptor 4, Colorectal neoplasms, Systematic review, Humans

## Abstract

**Purpose:**

Toll-like receptor 4 (TLR4) is increasingly recognized for its ability to govern the etiology and prognostic outcomes of colorectal cancer (CRC) due to its profound immunomodulatory capacity. Despite widespread interest in TLR4 and CRC, no clear analysis of current literature and data exists. Therefore, translational advances have failed to move beyond conceptual ideas and suggestions.

**Methods:**

We aimed to determine the relationship between TLR4 and CRC through a systematic review and analysis of published literature and datasets. Data were extracted from nine studies that reported survival, CRC staging and tumor progression data in relation to TLR4 expression. Primary and metastatic tumor samples with associated clinical data were identified through the Cancer Genome Atlas (TCGA) database.

**Results:**

Systematic review identified heterogeneous relationships between TLR4 and CRC traits, with no clear theme evident across studies. A total of 448 datasets were identified through the TCGA database. Analysis of TCGA datasets revealed TLR4 mRNA expression is decreased in advanced CRC stages (*P* < 0.05 for normal vs Stage II, Stage III and Stage IV). Stage-dependent impact of TLR4 expression on survival outcomes were also found, with high TLR4 expression associated with poorer prognosis (stage I vs III (HR = 4.2, *P* = 0.008) and stage I vs IV (HR = 11.3, *P* < 0.001)).

**Conclusion:**

While TLR4 mRNA expression aligned with CRC staging, it appeared to heterogeneously regulate survival outcomes depending on the stage of disease. This underscores the complex relationship between TLR4 and CRC, with unique impacts dependent on disease stage.

**Supplementary Information:**

The online version contains supplementary material available at 10.1007/s00432-022-04199-4.

## Introduction

Colorectal cancer (CRC) remains one of the most prevalent cancer diagnoses worldwide, with incidence rates in the United States of America of 37.8 per 100,000 (National Cancer and Institute: Surveillance 2021). This places CRC as the fourth most common cancer in western populations (Australian Institute and of Health and Welfare 2020; National Cancer and Institute: Surveillance 2021) which when coupled with its high mortality rates, cements this disease as a major healthcare burden. While significant advances have been made in identifying high level risk factors for CRC, heterogeneity in tumor progression and treatment response continues to challenge the understanding of its etiology (Buikhuisen et al. 2020). Few factors remain significant when traditional, largely unmodifiable risk factors (e.g. age, sex) are adjusted for, pointing to complex mechanisms governing tumor microenvironment which dictate growth trajectory and vulnerability to anti-cancer therapy (Buikhuisen et al. 2020).

The tumor microenvironment is a complex system of molecular and cellular components, produced by both host and tumor (Wang et al. 2018). The microenvironment’s contribution to prognosis and clinical outcome has proven controversial, although evidence supports both beneficial and inhibitory roles. For example, the microenvironment facilitates immune invasion and destruction of tumor tissue (Fang et al. 2014). In contrast, it also contributes to tumor development, cancer cell survival and treatment resistance (Zhao et al. 2019). Irrespective of this complexity, it is clear that infiltration of peripheral immune cells into the tumor microenvironment is related to CRC progression and prognosis. A 2019 study using the cancer genome atlas (TCGA) and gene expression omnibus (GEO) databases reported that M_0_ macrophages, M_1_ macrophages and CD4^+^ memory T cells were more abundant in CRC tissue compared to healthy tissues (*P* < 0.02) (Ge et al. 2019). Furthermore, higher infiltration of M_1_ macrophage populations in CRC tissue correlated with lower participant survival (*P* = 0.04) (Ge et al. 2019). This underscores the involvement of the host immune system in CRC.

In light of the strong immune-mediated mechanisms that appear to be linked with CRC etiology and treatment response, there has been substantial interest in the potential role of the innate immune surveillance protein, toll-like receptor 4 (TLR4). TLR4 is a pattern recognition receptor, which upon activation, initiates a strong inflammatory response (Takeda and Akira 2004). TLR4 requires the accessory proteins myeloid differentiation factor 2 (MD-2) and cluster of differentiation 14 (CD14) to efficiently bind to ligands including, LPS, heat shock proteins (Hsp70 and Hsp90) and high-mobility group protein I (HMGBI) (Cheng et al. 2015). TLR4 signaling is vital to intestinal homeostatic maintenance, as previously reviewed (Bruning et al. 2021). TLR4 is notably upregulated in the intestine under inflammatory states including in people with ulcerative colitis, and this is further linked to ulcerative colitis-associated CRC risk and development (Fukata et al. 2007). Furthermore, genetic variants of *TLR4* (rs10116253, rs192791 1, rs7873784) have been linked to CRC (Huang et al. 2018a).

TLR4 is expressed on a range of different cell types within the tumor microenvironment, including dendritic, stromal, macrophage and epithelial cells (Li J et al. 2017). The importance of site-specificity of TLR4 expression in healthy and diseased states, including CRC, is well documented (Bruning et al. 2021). Pre-clinical CRC models indicate that TLR4 has both pro- and anti- tumor roles, with expression sites being a possible differentiating factor between whether TLR4 aids in cancer destruction or survival (Li et al. 2017). To add further complexity, TLR4 has also been identified to modulate toxicity following cancer therapy, including diarrhea and pain (Wardill et al. 2016). As such, it is currently unclear whether TLR4 is beneficial, or, potentially harmful in the CRC microenvironment, and whether it is a rationale target for intervention. We therefore aimed to systematically review current published evidence and datasets to crystalize the relationship between TLR4 and CRC staging, treatment toxicity and survival.

## Methods

### Search strategy, study selection and data retrieval

PubMed, Cochrane Library and Embase were searched between January and February 2022 for peer-reviewed journal publications using keywords listed in Supporting Information Table 1 and were screened for inclusion based on specific criteria; original research, clinical trials and studies conducted between 2010 and 2021; archival human tissue; CRC; participant survival; tumor recurrence; prognosis; toxicity; and TLR4 expression. Exclusion criteria included: animal models; cell lines; and cancer types other than CRC. Eligible publications were reviewed with the following data being extracted manually by two independent authors (EEC, JKC) using a computer-based template: sample size; CRC stage; chemotherapy treatments; participant demographics; type of TLR4 analysis; TLR4 specific outcomes (including expression rates and site-specificity); survival data (overall survival (OS), progression-free survival (PFS) or disease-free survival (DFS)); and tumor progression data. Summary outcomes are presented in Table 1.

**Table 1:** Summary of studies investigating impacts of TLR4 expression on human CRC clinical outcome

Author (Date)	Archival/ clinical study	Sample size (*n*)	CRC stage	Anti-cancer treatments	TLR4 analysis	Site-specific TLR4 (Y/N)	Survival outcomes (Y/N)	Type of survival	Cancer recurrence (Y/N)	Key findings
Cammarota et. al. (2010)	Archival	132	Mixed stage, stages I–IV	NR	IHC	Y	Y	DFS	Y	- TLR4 cells = grade of dysplasia - % of TLR4 + cells in the tumor stromal compartment = DFS and later relapse compared to % of TLR4 + cells in the stromal compartment (RR 2.36; log rank chi-square 4.25, *P* < 0.05)
Tesniere et. al. (2010)	Clinical trial	668	Non-resectable metastases of colorectal adenocarcinoma and Stage II non-metastatic CRC	LV5FU2 followed by FOLFOX6 to FOLFOX6 and by FOLFIRI, or, surgical removal of the tumor	PCR *TLR4*	N	Y	PFS, or, progression 5 years after diagnosis	Y	- WT *TLR4* allele = PFS (HR: 0.73; CI = 0.53–1.00; *P* < 0.05) and OS (HR = 0.72; CI = 0.52–1.01); *P* = 0.05), compared to loss-of-function *TLR4* allele following treatment
Wang et. al. (2010)	Clinical trial	138	Mixed stage, stages I–IV	Surgery, chemotherapy and/or radiation treatment details NR	IHC	N	Y	DFS and OS	N	- TLR4 = 5-year DFS (HR (95% CI) 1.62 (0.87–2.99), *P* = 0.1213) and 5-year OS (HR (95% CI) 2.17 (1.15–4.07), *P* = 0.015) - TLR4 + MyD88 = 5-year DFS (HR (95% CI) 2.25 (1.27–3.99) *P* = 0.0053) and 5-year OS (HR (95% CI) 2.97 (1.64–5.38) *P* = 0.0003) - TLR4 expression was significantly associated with liver metastasis (*P* = 0.0001) - Co-expression of TLR4/MyD88 was significantly associated with vascular invasion (*P* = 0.0186), liver metastasis (*P* = 0.0002), and TNM stage (*P* = 0.0036)
Eiro et. al. (2013)	Clinical trial	104	Resectable, mixed stage, tumor stages I–IV	Surgery, varying chemotherapy and radiation treatments throughout sample population	IHC	Y	Y	OS	Y	- TLR4 expression by tumor cells = rate of tumor recurrence (*P* = 0.01) - TLR4 expression by fibroblasts = tumor recurrence (*P* = 0.019) - TLR4 expression by fibroblasts = OS (*P* = 0.022) - TLR4 expression by fibroblasts was an independent factor associated with relapse-free survival (*P* = 0.0001), and OS (*P* = 0.013)
Formica et. al. (2013)	Clinical trial	31	Mixed stage, stages I to III	FOLFIRI with bevacizumab	FC of TLR4 on neutrophils	Y	Y	PFS and OS	N	- No association between baseline or one-month post-treatment neutrophilic TLR4 expression and PFS or OS (*P* = 0.30 and *P* = 0.34 respectively)
Sussman et. al. (2014)	Archival	279	Mixed stage, stages I–IV	NR	IHC	Y	Y	OS	N	- No difference in TLR4 stromal staining and OS (*P* = 0.16), no difference in epithelial TLR4 staining and OS (*P* = 0.11) - TLR4 tumor stroma intensity score in stages 3 and 4 compared to stage 1 (Stage 1 = 2.80, Stage 2 = 3.24,Stage 3 = 4.36, Stage 4 = 3.75; *P* = NS, 0.0004, and 0.04, respectively) - TLR4 tumor epithelium intensity score for stages 2 and 3 compared to stage 1 (Stage 1 = 0.17, Stage 2 = 0.64, Stage 3 = 0.64, Stage 4 = 0.92; *P* = 0.01, 0.002, and NS, respectively)
Gray et. al. (2019)	Clinical trial	4877	Mixed stages inclusive of stage II, stage III and stable or responding metastatic CRC	SCOT trial (ISRCTN59757862): oxaliplatin-based adjuvant chemotherapy COIN trial (ISRCTN27286448): cetuximab added to oxaliplatin-based chemotherapy	PCR *TLR4*	N	Y	DFS and OS	N	- SCOT trial: no statistically significant association of any *TLR4* SNP and OS or DFS - COIN trial: no statistically significant association of either *TLR4* SNP with OS or DFS
Zhang et. al. (2019)	Clinical trial	94	Advanced stage, stages II and III	Standard 5-Fu-based adjuvant chemotherapy after radical surgery	WB, IHC	N	Y	DFS	Y	- Fn (*P* = 0.028) and BIRC3 expression (*P* = 0.046) correlated with DFS - TLR4 expression was independent of DFS; TLR4 was not a factor in univariate or multivariate cox regression analyses for DFS - TLR4 (*P* = 0.036) and BIRC3 (*P* = 0.008) resulted in recurrence
Wong et. al. (2021)	Clinical trial	46	Mixed stage, stages III–IV	Irinotecan monotherapy or in combination with 5-Fu and IFL regimen	PCR *TLR4*	N	N	NR	N	- Participants with *TLR4* SNPs rs4986790, rs4986791 severe diarrhoea (50%) than wild-type homozygous (15%) - Participants with TLR4 SNPs presented any grade of diarrhea, contrasting with one half of the AA and CC WT groups (20 patients each, AG + GG, *P* = 0.012 vs. AA; and CT + TT, *P* = 0.012 vs. CC) that showed no signs of gastrointestinal toxicity - No impact of TLR4 polymorphisms on occurrence/severity of nausea

*LV5FU2* Calcium leucovorin, citrovorum factor, folinic acid; *FOLFOX6* folinic acid, fluorouracil, and oxaliplatin; *FOLFIRI* fluorouracil, leucovorin and irinotecan; *5-Fu* Fluorouracil, *IFL* irinotecan, folinic acid, and fluorouracil. *IHC* Immunohistochemistry, *PCR* polymerase-chain reaction, *FC* flow cytometry, *WB* western blot, *OS* Overall survival, *DFS* disease-free survival, *PFS* progression-free survival, *NR* No record (NR)

### TCGA clinical CRC cases database extraction and statistical analysis

RNA sequencing data and associated clinical metadata with a total of 512 samples in read counts (HTSeq-Counts) of CRC were obtained from the TCGA data portal (https://portal.gdc.cancer.gov/, accessed in December 2020). Data related to TLR4 mRNA expression, CRC staging and OS were extracted. TLR4 mRNA expression was dichotomized into high and low expression using the tertile cut point. The OS curve was constructed using Kaplan–Meier and log-rank test analysis, comparing high and low TLR4 expression groups for all cases and within each CRC stage. Statistical analyses were performed using GraphPad Prism 8.3.1 (GraphPad Software Inc., CA, USA) and R. studio 1.2.5033 (Inc., Boston, MA).

Multivariate analysis was also performed to determine whether mRNA expression was associated with OS in each tumor stage where variables included tumor stage (I: IV), sex and age. To avoid using potentially biased cut-points splitting low and high TLR mRNA expressing participant groups, a two sample t-test using continuous TLR4 mRNA expression values (with no cut-point required) compared mRNA expression between alive and deceased participants. Finally, TLR4 mRNA expression between normal tumor adjacent tissue and tumor samples from different stages were analyzed with a one-way ANOVA (normal vs stage I, stage II, stage III and stage IV).

## Results

**Fig. 1:**
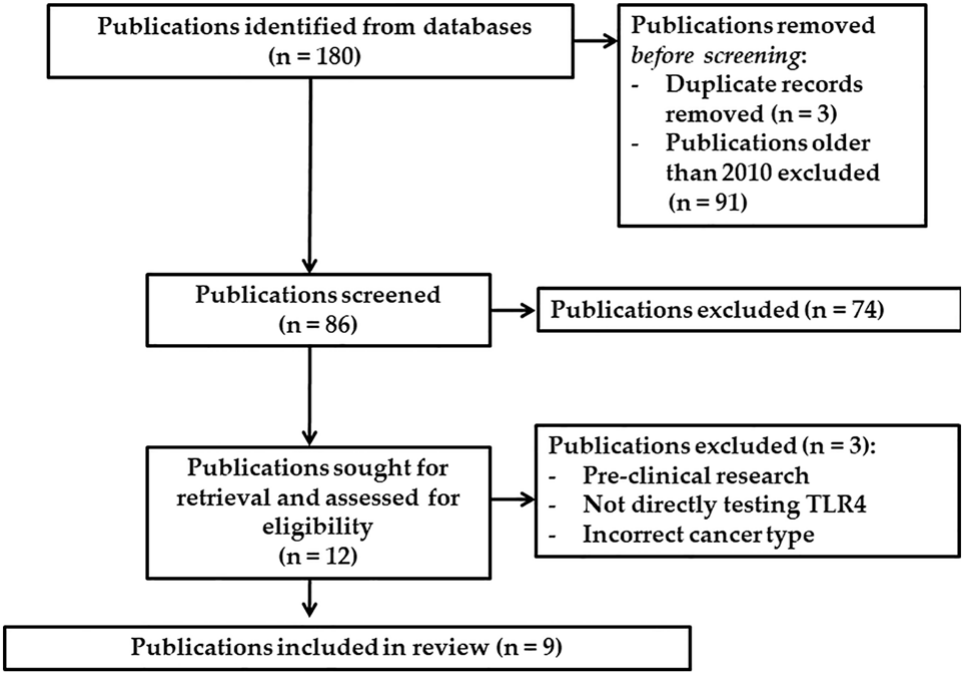
Flow diagram of literature search results for systematic review

180 publications were initially identified, with 9 meeting inclusion criteria for final analysis (Fig. 1). 6 publications were clinical trials with a combined participant total of 1081. The remaining 3 publications used archival tissue from previous clinical research. Only 2 publications analyzed advanced stage CRC (non-resectable tumor stage II–IV), whereas 7 publications included mixed analysis of varying CRC stage. Participant survival data was extracted from 8 publications, inclusive of DFS, PFS and OS dependent on individual study outcomes. Only 1 publication included data regarding toxicity in relation to TLR4 expression. Finally, CRC recurrence was analyzed in 3 publications. TLR4 expression in the publications was assessed using immunohistochemistry (5/9, all of which used different primary antibodies), polymerase chain reaction (PCR) (3/9) and flow cytometry (1/9). Only 4 publications included site-specific analysis of TLR4 expression in CRC (Table 1) (Cammarota et al. 2010; Eiro et al. 2013; Formica et al. 2013; Sussman et al. 2014). Of the 9 publications, 4 analyzed formalin fixed and paraffin embedded tissue blocks, 4 analyzed peripheral blood samples and 1 (Sussman et. al. 2014) analyzed tumor tissue microarray slides provided by the NCI Cancer Diagnosis Program (CDP).

### Impact of TLR4 genotype and expression on CRC survival

Of the 8 publications to report on CRC survival, one reported that wild-type (WT) *TLR4* genotype was beneficial to CRC participant survival rates (Tesniere et al. 2010). Metastatic CRC participants with the WT *TLR4* allele had higher PFS (hazard ratio (HR): 0.73; 95% confidence interval (CI) = 0.53–1.00; *P* < 0.05) and OS (HR = 0.72; 95% CI = 0.52–1.01; *P* = 0.05) compared with participants bearing the *TLR4* loss-of-function (Asp299Gly) variant post-oxaliplatin chemotherapy treatment (Tesniere et al. 2010). No differences in DFS among participants bearing the WT versus the variant *TLR4* alleles were observed.

In contrast, 2 publications suggested that increased TLR4 expression is detrimental to participant survival (Cammarota et al. 2010; Wang et al. 2010). Cammarota et al. found that in mixed stage CRC tissue, participants with lower TLR4 expression in the tumor stroma compartment had improved DFS compared to participants with higher TLR4 expression (risk ratio (RR) 2.36; log-rank chi-square 4.25, *P* < 0.05) (Cammarota et al. 2010). Furthermore, participants with pT_3_ adenocarcinoma with high TLR4 expression (over 50% positive cells) relapsed sooner (14 months) compared to participants with low TLR4 expression (40 months, RR 3.15; log-rank chi-square 4.03, *P* < 0.05) (Cammarota et al. 2010). This is supported by Wang and colleagues, who confirmed that CRC tissue displayed expression of TLR4 in 78 of 108 samples (72%), of which 22 displayed high TLR4 expression (Wang et al. 2010). In addition, increased TLR4 expression was associated with liver metastasis (*P* = 0.0015) and advanced tumor stage (stage IV) (*P* = 0.0197). Upon univariate analysis there was no difference in 5-year DFS rate for low versus high TLR4 expression, but OS was reduced with high TLR4 expression (HR (95% CI) 2.17 (1.15–4.07), *P* = 0.015) (Wang et al. 2010). However, this was not retained in multivariate analysis. In contrast, when samples exhibited high expression of both TLR4 and the adapter protein MyD88, DFS and OS were poorer (HR (95% CI) 2.11 (1.05–4.23) *P* = 0.0352) (Wang et al. 2010).

The conflicting nature of outcomes may be reflective of the lack of site-specific TLR4 investigations throughout human CRC research. Eiro and colleagues reported TLR4 expression by fibroblasts, not tumor cells themselves, was associated with a shortened OS of CRC participants (*P* = 0.022). Furthermore, TLR4 expression in fibroblasts was a significant and independent factor associated with DFS (*P* = 0.0001), and OS (*P* = 0.013) (Eiro et al. 2013).

Four publications reported that TLR4 expression does not impact upon CRC survival. Formica and colleagues found that in 31 metastatic CRC participants, neutrophil TLR4 expression at baseline, or 1-month post-chemotherapy, had no association with PFS (*P* > 0.05) (Formica et al. 2013). This is supported by Sussman and colleagues who, in *N* = 279, found no associated between TLR4 expression in stromal tissue and OS after correcting for both CRC stage and grade. Furthermore, epithelial TLR4 expression was also not associated with OS (Sussman et al. 2014).

More recently, Zhang and colleagues found that in an advanced CRC cohort (*N* = 94) post-standard Fluorouracil-based adjuvant chemotherapy and radical surgery, the measured level of TLR4 expression was independent of DFS; hence no impact of TLR4 on overall DFS (Zhang et al. 2019). In addition, TLR4 was not a significant factor in survival outcomes following univariate or multivariate analyses (Zhang et al. 2019). However, high amounts of Fusobacterium (*Fn*), an anaerobic bacterium known to activate the TLR4 pathway in CRC cells, correlated with poor DFS (*P* = 0.028) (Zhang et al. 2019). Finally, Gray and colleagues analyzed previously collected tissues from two large-scale clinical trials, the SCOT (ISRCTN59757862) trial and COIN (ISRCTN27286448) trial (Gray et al. 2019). Data generated from SCOT showed no association of any *TLR4* single nucleotide polymorphism (SNP) with survival (Gray et al. 2019). There was also no association of the *TLR4* SNP, rs867228, with DFS in cases with functional polymorphisms (Gray et al. 2019). Data from COIN showed no association of any tested *TLR4* SNP with OS by either log-rank test or univariate or multivariable Cox regression (Gray et al. 2019).

### CRC recurrence

Three publications reported on TLR4s contribution to CRC recurrence, with 2 publications identifying a detrimental role of TLR4 in CRC recurrence (Wang et al. 2010; Zhang et al. 2019). Wang and colleagues (2010) report upon 5 year follow-up of 108 mixed stage CRC participants, 53 participants had tumor recurrence (DFS rate: 49%), with participants exhibiting high expression of TLR4 and its accessory protein MyD88 displaying increased recurrence rates compared to those with low expression (TLR4 + MyD88 (low vs high) 5-year DFS HR (95% CI) = 2.25 (1.27–3.99) *P* = 0.0053) (Wang et al. 2010). Furthermore, participants with CRC and liver metastasis showed higher TLR4 and MyD88 expression versus CRC without liver metastasis (Wang et al. 2010). Among the 14 liver metastases obtained by hepatectomy, 12 were TLR4 positive and 6 showed a high expression (Wang et al. 2010). These findings are supported by Zhang and colleagues who showed high expression of TLR4 (*P* = 0.036) were more likely detected in participants with CRC recurrence, compared with participants without recurrence (Zhang et al. 2019).

In contrast, Eiro and colleagues observed that recurrence was dependent on the site of TLR4 expression, not its overall quantitative expression such that TLR4 expression by tumor cells was associated with a lower rate of recurrence in tumors from left colon/rectum compared to those from right colon/rectum (*P* = 0.028) (Eiro et al. 2013). Further, TLR4 expression by fibroblasts was associated with a high rate of recurrence (*P* = 0.0001) in left colon/rectum tumors (Eiro et al. 2013).

### Toxicity post-chemotherapy in participants with CRC

Only 1 publication investigated the role of TLR4 in relation to post-chemotherapy toxicity outcomes, including diarrhea and nausea. Wong and colleagues investigated a cohort of 46 advanced stage CRC (stage III–IV), treated with first cycle of irinotecan-based chemotherapy (irinotecan monotherapy or in combination with fluorouracil and leucovorin—IFL regimen) (Wong et al. 2021). Participants the variant *TLR4* SNPs rs4986790 and rs4986791 had more severe diarrhea (50%) compared to those without the variants (15%) (Wong et al. 2021). When looking at diarrhea of all severities, all participants (100%) with the variant *TLR4* SNPs developed diarrhea, compared to only 50% of those without the variants (20 participants each, rs4986790, *P* = 0.012 vs. rs4986791, *P* = 0.012).(Wong et al. 2021) There was no association with nausea (Wong et al. 2021).

## TCGA database results

### TLR4 expression differs due to cancer stage

**Fig. 2:**
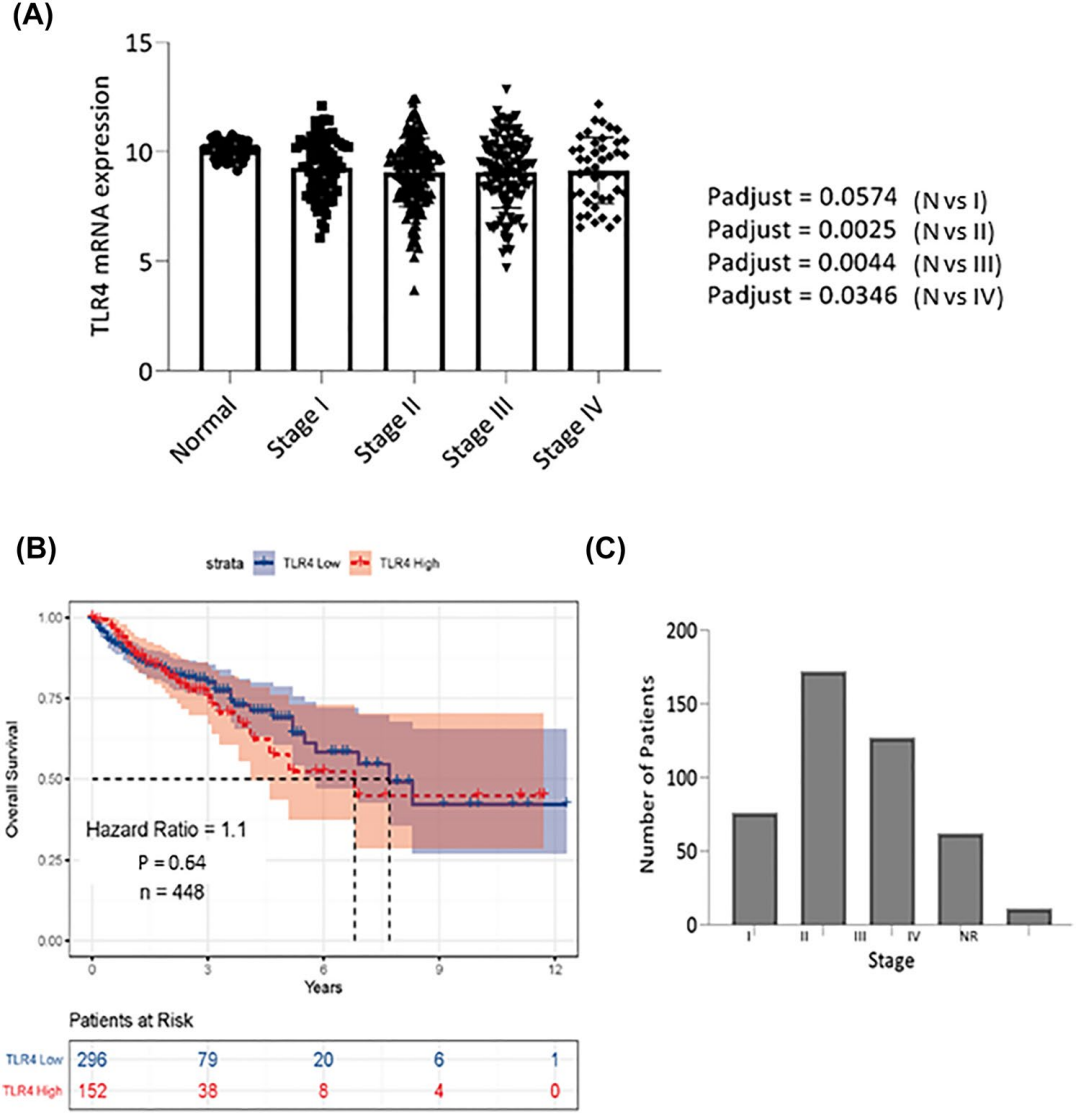
**A** Comparison of TLR4 expression between stage specific tumor and adjacent normal tissues from TCGA cohort. One-way ANOVA was performed by comparing solid tissue normal vs stage I, stage II, stage III, and stage IV participants. Statistical significance was represented as *P* < 0.05. **B**, **C** Assessment of TLR4 mRNA expression using the tertile cut-point. (B) Kaplan–Meier curves of overall survival (OS) in TCGA cohort. (C) Bar plot depicting the stage distribution of the cohort

Summary of participant clinical data is presented in supporting information Table 2. Although TLR4 expression was not statistically different between normal and stage I, significantly higher TLR4 expression was observed in normal tissues vs Stage II, Stage III and Stage IV (Fig. 2A).

### TLR4 expression is associated with survival in respect to tumor stage

**Fig. 3:**
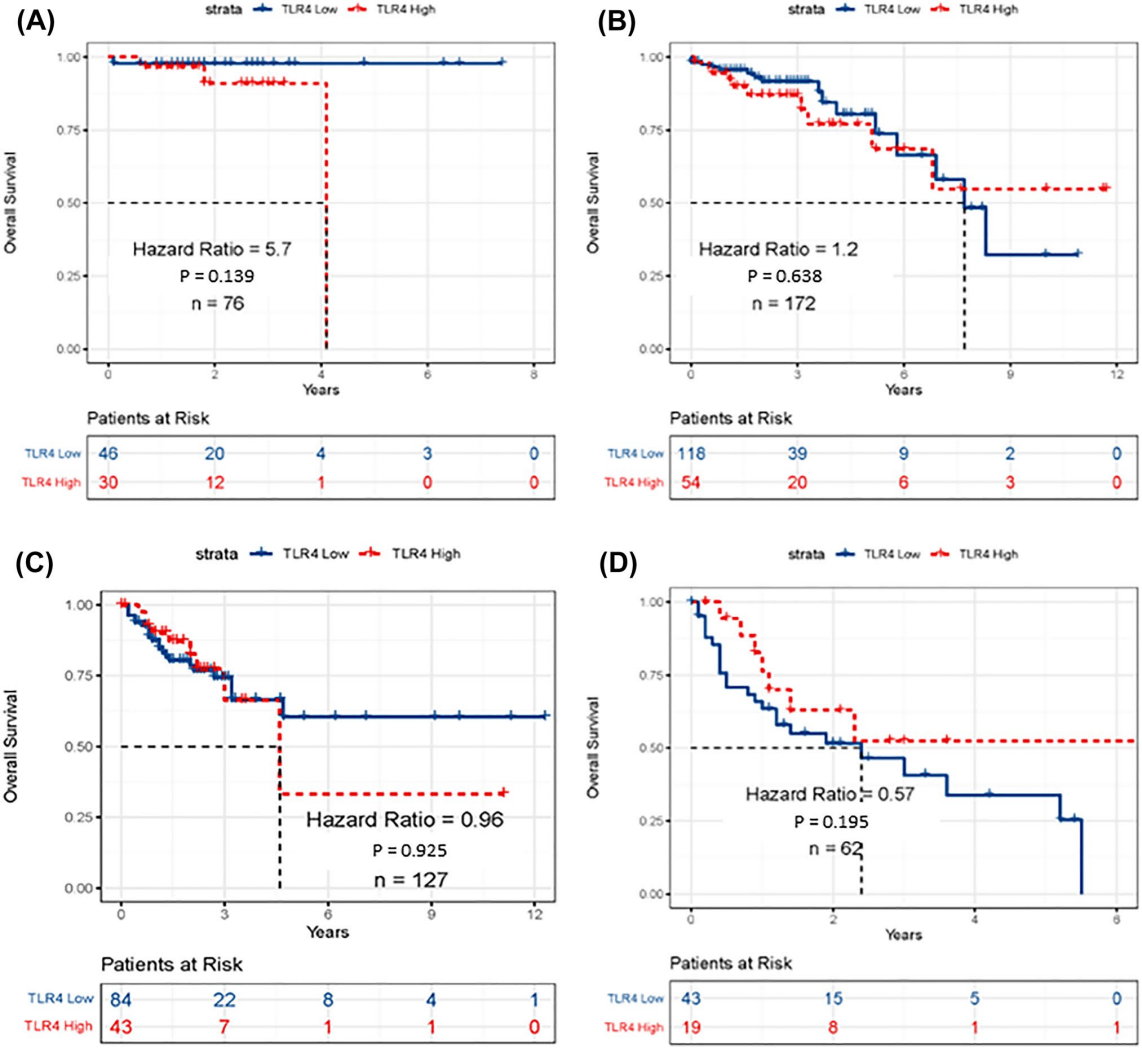
Assessment of TLR4 mRNA expression in stage specific CRC participants from TCGA cohort. **A** Kaplan–Meier curves depicting the OS in stage I participants **B** stage II participants, **C** stage III participants and **D** stage IV participants using the tertile cut point. No significant difference between groups

**Fig. 4:**
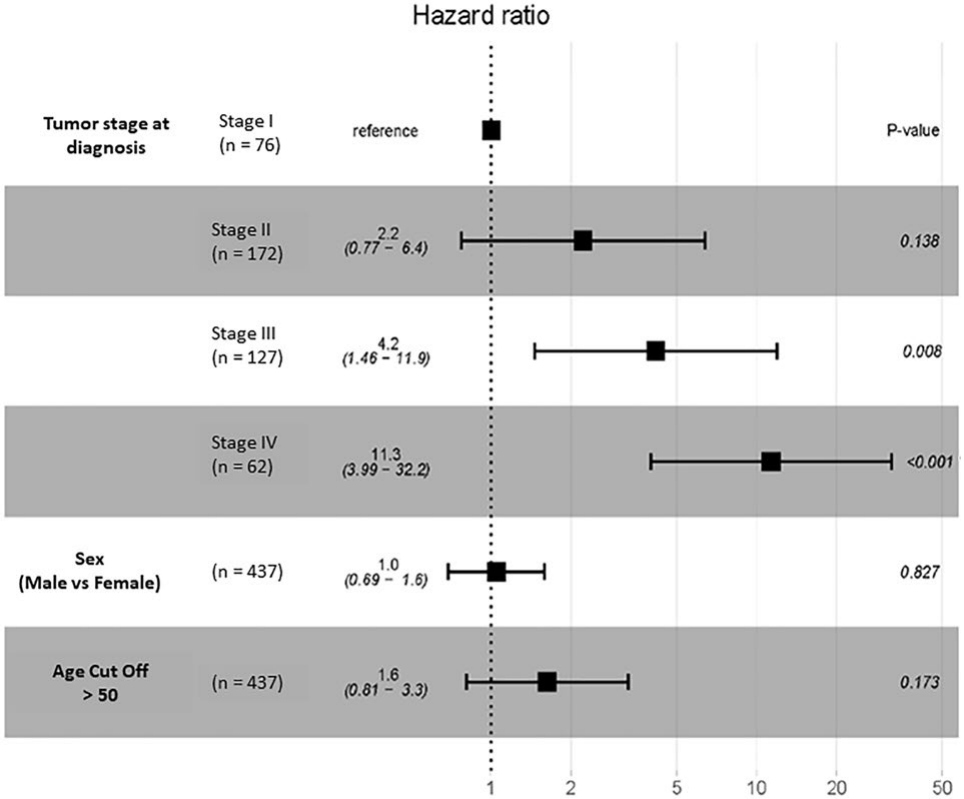
Forest plot of OS in stage specific participants. The tertile cut-point, the p-values and HRs with 95% CI derived for measurement of the cohorts from assessing the cut-point were shown. Statistical significance was represented as *P* < 0.05

**Fig. 5:**
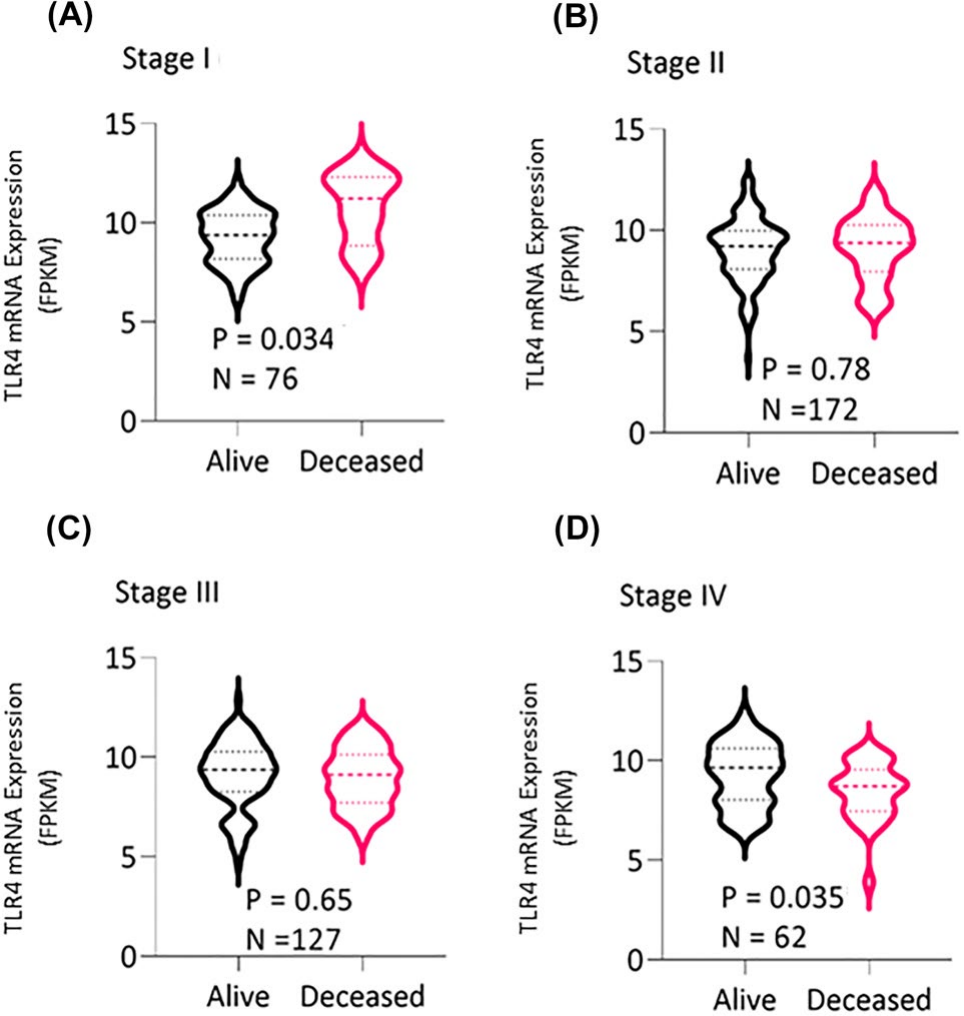
Comparison of TLR4 expression in Fragments per Kilobase of transcript, per Million mapped reads (FPKM) with respect to OS. Analysis of TLR4 expression using two sample t-test based on participants’ survival in **A** stage I, **B** stage II, **C** stage III, and **D** stage IV participants. Statistical significance was represented as *P* < 0.05

Number of participants per tumor stage is presented in Fig. 2C. OS of participants with CRC with respect to TLR4 expression (low vs high) was conducted. TLR4 expression was not a significant prognostic factor (HR = 1.1, *P* = 0.64) when all stages were combined (Fig. 2B) or compared between stages (Fig. 3). In contrast, multivariate analysis revealed high TLR4 expression prior to treatment conferred worse prognosis, with the strength of the effect increasing with tumor stage (stage I vs II (HR = 2.2, *P* = 0.138), stage I vs III (HR = 4.2, *P* = 0.008) and stage I vs IV (HR = 11.3, *P* < 0.001); Fig. 4). Sex and age had no impact on OS (Fig. 4). In stage I disease, those that were alive had lower TLR4 expression at diagnosis (*P* = 0.034). For all other stages TLR4 expression at diagnosis was higher in those still alive (*P* = 0.035) (Fig. 5).

## Discussion

TLR4 is an attractive target for controlling cancer development and optimizing treatment response due to its potent regulation of systemic immune responses. Our analysis exposes the significant heterogeneity in CRC outcomes linked with TLR4 expression. We have shown that TLR4 expression decreases with increasing CRC tumor stage at prognosis, and appears to have stage-dependent associations with participant outcomes. We highlight two novel findings related to high TLR4 expression in early- and late-stage CRC being; (1) in stage I CRC results in worse participant outcomes, and (2) in stage IV CRC results in improved participant outcomes. With TLR4 expression decreasing in higher grade CRC, this potential reduction of innate immune signaling may prove to be the causative mechanism behind unfavorable treatment responses and reduced survival.

TLR4 expression relative to tumor stage is well documented in the literature (Li et al. 2019; Omrane et al. 2014). These patterns of TLR4 expression reflect its core physiological mechanism of inducing inflammation, a process known to be carcinogenic. Our data showed a significant decrease in TLR4 expression in later stage CRC (stages II–IV) compared to normal tissue. This decrease in TLR4 expression was not found in stage I tumors, suggesting that the slightly higher TLR4 expression in early CRC may align with the well-defined concept that inflammatory processes are involved in the early development of CRC (Karin and Greten 2005). However, our analysis did show that non-tumor comparative tissue had the highest TLR4 expression. As this tissue was primarily collected from adjacent tissue in the same participants, systemic inflammatory responses may have impacted on interpretation. The finding that TLR4 expression decreases with tumor growth is also consistent with the current understanding of tumor development, with tumors often adapting to evade immune detection and control. Activation of the receptor, programmed death 1 (PD-1), has been found to inhibit immune control of tumor growth, with the PD-1 ligand, PD-L1, being significantly upregulated in solid tumors like CRC (Hino et al. 2010). Therefore, this upregulation of PD-L1 is suggested to play a crucial role in the tumors ability to evade host immune system (Dong et al. 2002). This is of particular interest in the context of TLR4 research, as PD-L1 has also been shown to block the cytolytic activity of PD-1+ tumor infiltrating CD4^+^ and CD8^+^ T cells, which are reliant on dendritic cell -TLR4 interaction (Brahmer et al. 2012; Fife et al. 2009). In addition, Xiao et. al. (2016) reported that inhibition of TLR4 signaling via a blocking antibody significantly reduced the number of PD-1+ B cells in human hepatoma tissues, where PD-1+ B cell populations promoted cancer growth (Xiao et al. 2016). Furthermore, Huang (2018) found that improvement in clinical outcome is resultant of cytosolic HMGB1 triggering dendritic cell maturation through TLR4 activation, whereby consequently recruiting PD-1+ tumor-infiltrating lymphocytes to the tumor site (Huang et al. 2018b). These findings highlight the importance of TLR4 to this particular tumor kill pathway and outlines the importance for TLR4 expression for improved clinical outcomes of people living with CRC.

While our findings suggest a likely relationship between TLR4 expression and tumor stage, the relationship between TLR4 and long-term outcome was less clear cut in both our systematic review and genetic analyses. When looking at all tumor stages, there was no significant impact on OS in low vs high TLR4 expressing tumors. This contradicts existing data, as a metanalysis of 212 people living with CRC found that high TLR4 expression associated with a significantly reduced OS and poorer prognosis (HR (95% CI) 2.30 (1.41, 3.75), *P* = 0.001) (Hao et al. 2018). However, this analysis did not classify the cohort based on CRC stage which may have masked some findings and increased bias towards advanced stage disease. While our initial analyses showed no effect of TLR4 expression on OS, analysis of this relationship within specific tumor stages revealed that TLR4 may in fact have an impact but, in a stage-specific manner. Specifically, we showed that TLR4 expression in Stage IV disease was higher in tumors from people still alive compared to those that were not. While we weren’t able to show this in our longitudinal OS analyses, this may reflect the lack of power when breaking down our cohort of 488 into specific stages.

This heterogeneity in how TLR4 may act to regulate overall survival for Stage I vs Stage IV disease is likely to reflect the differences in how these disease stages are treated. Stage I disease is almost always treated with surgery, but no cytotoxic therapy, whereas stage IV disease will certainly contain cytotoxic therapy. TLR4 is considered to exert its impact on treatment outcomes via its ability to modulate immunogenic cell death (Fang et al. 2014; Kroemer et al. 2013). Immunogenic cell death acts in concert with direct cytotoxicity, and collectively results in more thorough tumor clearance, and thus long-term survival. As such, higher TLR4 expression would theoretically confer a larger immune response and thus better response in late-stage CRC. This is supported by the Isambert et al. study (2013) which found that increased activation of TLR4 via a lipid A analogue (OM-174) enhanced inflammatory anti-tumor response in metastatic CRC and improved clinical outcomes (Isambert et al. 2013). Furthermore, data from Huang and colleagues (2018) showed improved DFS in people living with late-stage rectal cancer with increased activation of TLR4 via HGMB1 binding (Huang et al. 2018b).

Despite new interpretation of stage-specific roles of TLR4, we must acknowledge some limitations of our approach. Firstly, the studies included within the literature review were varied, often with low sample sizes and differing approaches to measuring TLR4 expression. Furthermore, our genetic analysis relied on previously collected data and exhibited low power when analyzing within the specific CRC stages. It is also important to acknowledge that we relied solely on TLR4 tumor-expression data; whereas evidence from pre-clinical work suggests expression of TLR4 in host tissues (typically non-cancerous) may be critical in setting immune tone of host and thus response (Li et al. 2017). Nonetheless, our findings indicate a general trend towards higher TLR4 expression being associated with favorable OS outcomes in stage IV CRC suggesting its ability to induce immunogenic cell death is critical in CRC prognosis.

## Supplementary Information

Below is the link to the electronic supplementary material.

Supplementary file1 (DOCX 15 KB)

Supplementary file2 (DOCX 14 KB)
