# Supplementary material for: Contribution of TLR4 to colorectal tumor microenvironment, etiology and prognosis
Source: J Cancer Res Clin Oncol. 2022 Jul 16;149(7):3009–21. doi: 10.1007/s00432-022-04199-4 (PMC10314848; doi:10.1007/s00432-022-04199-4)
Supplement: Supplementary file 1 — Supplementary file1 (DOCX 15 KB) [file 432_2022_4199_MOESM1_ESM.docx]

| Database | Date Accessed | Search Strategy | Identified Literature |
| --- | --- | --- | --- |
| PubMed | January 2022 – February 2022 | “Toll-Like Receptor 4”[mh] OR TLR4[tiab] OR Toll 4 Receptor[tiab] OR Toll Like Receptor 4[tiab] AND “Antineoplastic Protocols”[mh] OR chemotherap*[tiab] NOT ("Animals"[Mesh] NOT ("Animals"[Mesh] AND "Humans"[Mesh])) and (((((“Toll-Like Receptor 4”[mh] OR TLR4[tiab] OR Toll 4 Receptor[tiab] OR Toll Like Receptor 4[tiab]) AND (“Antineoplastic Protocols”[mh] OR chemotherap*[tiab])) AND (colon OR colo* OR bowel)) AND (cancer OR cancer* OR tumour* OR tumor)) NOT (("Animals"[Mesh] NOT ("Animals"[Mesh] AND "Humans"[Mesh])))) AND (((("Toll-Like Receptor 4"[mh] OR TLR4[tiab] OR Toll 4 Receptor[tiab] OR Toll Like Receptor 4[tiab]) AND ("Antineoplastic Protocols"[mh] OR chemotherap*[tiab])) AND (colon OR colo* OR bowel)) AND (cancer OR cancer* OR tumour* OR tumor)) NOT (("Animals"[Mesh] NOT ("Animals"[Mesh] AND "Humans"[Mesh]))). | 36 individual publications   - 34 when within 2010 – 2021 timeframe - only 2 conformed with eligibility criteria |
| Cochrane Library | January 2022 – February 2022 | “Trials AND TLR4 AND cancer and colorectal” | 5 individual publications   - No publications conformed with eligibility criteria |
| Embase | January 2022 – February 2022 | “Toll Like Receptor 4”/de OR TLR4:ti,ab OR “Toll 4 Receptor*”:ti,ab OR “Toll Like Receptor 4”:ti,ab AND “Antineoplastic Protocols”:ti,ab OR chemotherap*:ti,ab OR chemotherapy/exp NOT ([animals]/lim NOT [humans]/lim) and ('toll like receptor 4'/de OR tlr4:ti,ab OR 'toll 4 receptor*':ti,ab OR 'toll like receptor 4':ti,ab) AND ('antineoplastic protocols':ti,ab OR chemotherap*:ti,ab OR 'chemotherapy'/exp) AND (colon OR colorectal OR bowel OR intestine) NOT ([animals]/lim NOT [humans]/lim) AND (cancer OR tumour OR tumor OR 'malignant neoplasm').  and  ((Toll Like Receptor 4 or TLR4 or Toll 4 Receptor* or Toll Like Receptor 4) and (Antineoplastic Protocols or chemotherap* or chemotherapy) and (toll like receptor 4 or tlr4 or toll 4 receptor* or toll like receptor 4) and (antineoplastic protocols or chemotherap* or 'chemotherapy) and (colon or colorectal or bowel or intestine) and (cancer or tumour or tumor or malignant neoplasm)).af | 139 individual publications   - 52 when within 2010 – 2021 timeframe - 3 duplicates removed - 4 articles removed due to ineligibility with inclusion and exclusion criteria - Only 7 conformed with eligibility criteria |

Supporting information Table 1: literature database search strategies and publication results.
