# Supplementary material for: Contribution of TLR4 to colorectal tumor microenvironment, etiology and prognosis
Source: J Cancer Res Clin Oncol. 2022 Jul 16;149(7):3009–21. doi: 10.1007/s00432-022-04199-4 (PMC10314848; doi:10.1007/s00432-022-04199-4)
Supplement: Supplementary file 2 — Supplementary file2 (DOCX 14 KB) [file 432_2022_4199_MOESM2_ESM.docx]

| **Characteristic** | **N (%)** |
| --- | --- |
| **Age** |  |
| Median (Range) | 68.00 (31-90) |
| **Sex** |  |
| Male | 239 (53) |
| Female | 209 (47) |
| **CRC stage at prognosis** |  |
| I | 76 (17) |
| II | 172 (38) |
| III | 127 (28) |
| IV | 62 (14) |
| not reported | 11 (2) |
| **TLR4 (CPM)** |  |
| Median (Range) | 9.23 (3.7-2.8) |
| **Site of resection or biopsy** |  |
| Ascending colon | 87 (19) |
| Cecum | 90 (20) |
| Colon, NOS | 98 (22) |
| Descending colon | 16 (4) |
| Hepatic flexure of colon | 15 (3) |
| Recto sigmoid junction | 8 (2) |
| Sigmoid colon | 110 (25) |
| Splenic flexure of colon | 5 (1) |
| Transverse colon | 19 (4) |
| **Vital Status** |  |
| Alive | 352 (79) |
| Deceased | 96 (21) |
| **History of neo-adjuvant treatment** |  |
| Yes | 0 (0) |
| No | 448 (100) |

**Supporting Information Table 2:** Summary of clinical participant data (n = 448) from TCGA analyses. Data are n (%) unless otherwise stated. Data extracted on 6/6/2020.
